# Supplementary material for: Splice-Junction-Based Mapping of Alternative Isoforms in the Human Proteome
Source: Cell Rep. Author manuscript; Available in PMC 2020 Jan 15. (PMC6961840; doi:10.1016/j.celrep.2019.11.026)
Supplement: 3 [file NIHMS1546469-supplement-3.zip › DF2/PXD0010154/Liver-221-Q96RU2-SGVEAALSEAFHEEYSR.pdf]

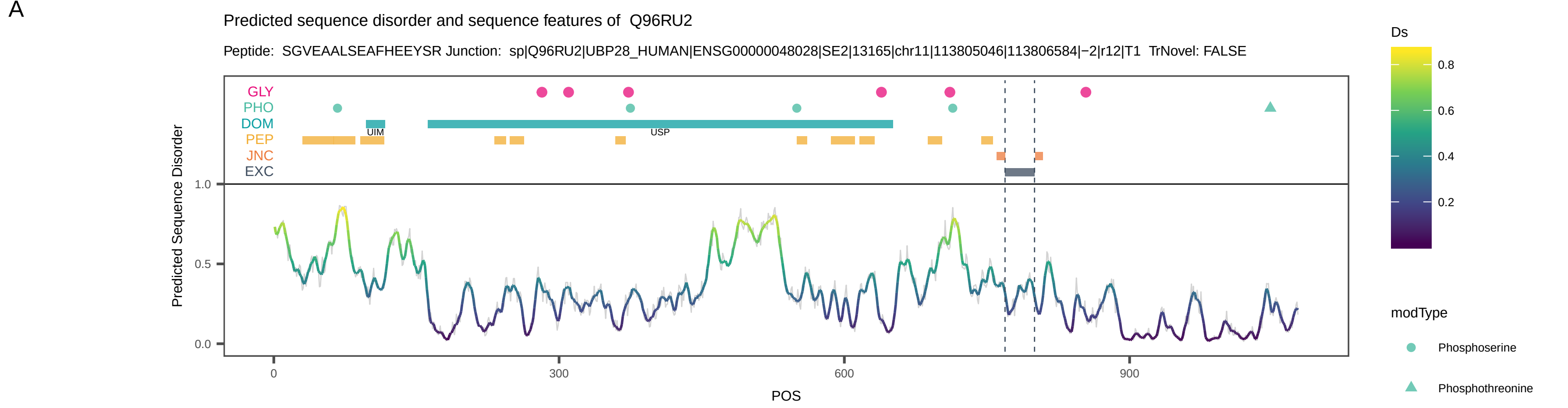
A
Predicted sequence disorder and sequence features of Q96RU2
Peptide: SGVEAALSEAFHEEYSR Junction: sp|Q96RU2|UBP28_HUMAN|ENSG00000048028|SE2|13165|chr11|113805046|113806584|−2|r12|T1 TrNovel: FALSE
GLY
PHO
DOM
PEP
JNC
EXC
UIM
USP
Predicted Sequence Disorder
1.0
0.5
0.0
0
300
600
900
POS
Ds
0.8
0.6
0.4
0.2
modType
Phosphoserine
Phosphothreonine

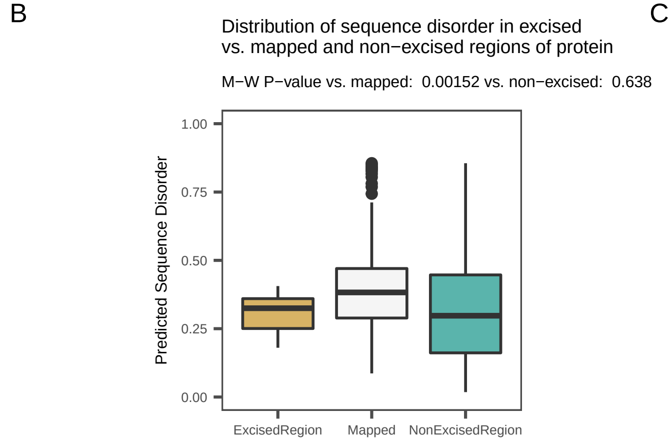
B
Distribution of sequence disorder in excised
vs. mapped and non−excised regions of protein
M−W P−value vs. mapped: 0.00152 vs. non−excised: 0.638
Predicted Sequence Disorder
1.00
0.75
0.50
0.25
0.00
ExcisedRegion
Mapped
NonExcisedRegion

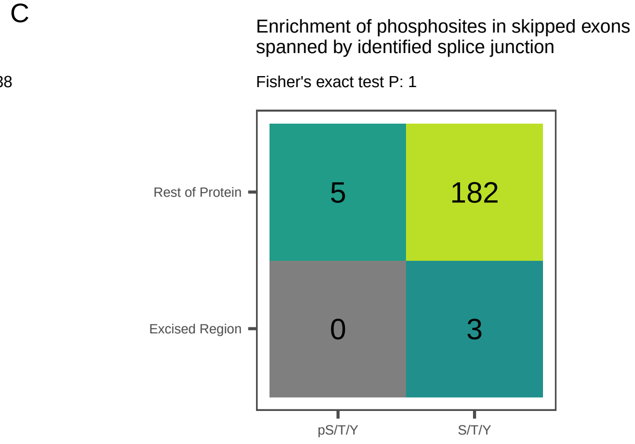
C
Enrichment of phosphosites in skipped exons
spanned by identified splice junction
Fisher's exact test P: 1
Rest of Protein
Excised Region
5
182
0
3
pS/T/Y
S/T/Y
